# Risks and Benefits of Web-Based Patient Narratives: Systematic Review

**DOI:** 10.2196/15772

**Published:** 2020-03-26

**Authors:** Daniel Drewniak, Andrea Glässel, Martina Hodel, Nikola Biller-Andorno

**Affiliations:** 1 Institute of Biomedical Ethics and History of Medicine University of Zurich Zurich Switzerland; 2 Institute of Health Sciences Zurich University of Applied Sciences Winterthur Switzerland

**Keywords:** patient experiences, personal narratives as topic, systematic review

## Abstract

**Background:**

Patient narratives are illustrative, individual accounts of patients’ experiences with certain health conditions. Web-based patient narratives have become widely available on the internet and in social media, as part of electronically available patient decision aids or Web-based databases. In recent years, the role of patient narratives as a source of information, insight, and support for both health care users and providers has increasingly been emphasized. Although the potential impact of patient stories has high immediate plausibility, it is of interest to know if this impact can be captured in quantitative studies.

**Objective:**

This review aimed to evaluate whether research-generated Web-based patient narratives have quantifiable risks or benefits for (potential) patients, relatives, or health care professionals.

**Methods:**

We searched the following databases from August 2017 to March 2019: Medical Literature Analysis and Retrieval System Online, PsycInfo, Sociological Abstracts, Web of Science, and EMBASE. Titles and abstracts of the retrieved studies were reviewed and assessed for the inclusion and exclusion criteria. Papers were included if they studied the following: (1) (potential) patients, relatives, or health care professionals; (2) the effects of Web-based patient narratives that were generated scientifically (eg, through qualitative research methods); and (3) were quantitative studies. Furthermore, 2 authors independently performed an assessment of the quality of the included studies using a validated checklist.

**Results:**

Of 4226 documents, 17 studies met the inclusion criteria. The studies investigated 10 different sources of Web-based patient narratives. Sample sizes ranged from 23 to 2458. The mean score of the quality assessment was 82.6 (range 61-100). Effects regarding five different purposes were identified as follows: provide information, engage, model behavior, persuade, and comfort. We found positive effects in every category and negative effects in one category (persuade). Several of the reported effects are rather small or were identified under specific experimental conditions.

**Conclusions:**

Patient narratives seem to be a promising means to support users in improving their understanding of certain health conditions and possibly to provide emotional support and have an impact on behavioral changes. There is limited evidence for beneficial effects on some outcomes. However, narratives are characterized by considerable heterogeneity and the investigated outcomes are hardly comparable with each other, which makes the overall judgment difficult. As there are numerous possible measures and purposes of narratives, quantifying the impact of Web-based patient narratives remains a challenge. Future research is needed to define the optimal standards for quantitative approaches to narrative-based interventions.

## Introduction

### Background

In their recent report, the *Lancet Global Health Commission* calls for an improved integration of patient experiences in the evaluation of health care systems, including experiences about competent care, health care utilization, or confidence in the health care system [1]. Such experiences can be collected by using tools such as patient satisfaction surveys. Although quantitative data about patient experiences are essential measures for the accountability and improvement of health care systems, they fall short of capturing a more comprehensive picture of how patients experience health care encounters or illnesses [1,2].

Patient narratives are illustrative accounts of individual patients’ experiences with a certain illness [3] and are available on social media sites, in patient decision aids, and on databases such as the *Database of Individual Patients’ Experiences* (DIPEx). There is neither a clear definition of what constitutes a narrative nor any guidance on the length or content [4], which may lead to conflicting research results about the effects of patient narratives because of insufficient operationalization of the term [3].

Patient narratives are a promising tool that can support people in coping with their illness [5], serve as a resource for preparing health care decisions [6], or help identify questions for physicians [7]. Characteristically, narratives can retrospectively structure actions in ways that convey perceived causality; they are nonlinear and powerful in making sense of complex, emergent phenomena [8]. Furthermore, stories transport images and emotions, which makes them evocative and memorable. Most people recall stories better than statistical information expressed in graphs or numbers [2,8].

Several qualitative studies report that illness narratives enjoy high acceptance among other patients [9]. Furthermore, positive effects of personal health and illness experiences, including improvements in decision making [10,11] or addressing information needs [12], were identified in qualitative studies. Narratives have a high potential to add unknown insights into patient-focused issues, which can only be provided by a person who has the respective lived experience. For example, as a World Health Organization report states, “qualitative methods help to present narratives that broadly reflect the gendered social norms about parent-child relations. They also provide ‘lived experiences’ from ageing populations about how satisfied they are with the life they have lived” [2].

On the contrary, there are also serious concerns about the use of patient narratives because they are powerful message formats [13] and are suspected to override statistical information [14,15]. The concern is that patients’ decision-making regarding treatment options could be based on personal experiences of a few, whereas statistical data remain largely ignored [2,14,15]. Furthermore, patients’ experiences presented on the Web may contain unbalanced or misleading messages, which may lead to a manipulation of choices in favor of a particular health care option [16]. A study among mothers of children with genetic disorders, eg, found that several parents put more trust on online communities than on their physicians [17]. Such findings are especially problematic when stories in such communities are biased.

In recent years, internet platforms, patient blogs, and fora have become important means for individuals to seek information relevant to health, including information describing how other individuals live with illnesses. Such websites often provide biomedical information but lack information on wider experiences [18] or the experiences are not systematically collected, analyzed, and presented [19]. Therefore, in this review, we focused on studies that used established scientific methodologies to elicit the stories [8,20].

### Objectives

This systematic review aimed to evaluate whether research-generated Web-based patient narratives have quantifiable risks or benefits for patients, relatives, or health care professionals. Patient narratives are understood as immediate personal experience reports.

## Methods

### Search Strategy

This review was conducted in accordance with the Preferred Reporting Items for Systematic Reviews and Meta-Analysis guidelines [21]. To identify relevant studies, the databases Medical Literature Analysis and Retrieval System Online, PsycInfo, Sociological Abstracts, Web of Science, and EMBASE were searched from August 2017 to March 2019. A search term was developed and was adjusted to the different databases. The search terms were tested and evaluated by the study team. In addition, the search strategy was discussed and evaluated with a member of Cochrane Switzerland and with an employee from the University library who specialized in systematic reviews. The search terms were adjusted based on the discussion and recommendations. The search terms consisted of the following: [Narration: narration, personal narratives, narrative medicine, anecdot*, testimonial*] + [Databases: internet, bibliographic database, online, Web based] + [Participants: patient, health care personnel, relative*, caregivers] + [Study: Surveys and Questionnaires, controlled clinical trials, cohort studies] (Multimedia Appendix 1).

### Selection Criteria

Titles and abstracts of the retrieved studies were reviewed and assessed for inclusion and exclusion criteria independently by all members of the study team (DD, AG, MH, and NB). Researchers were trained in applying the predefined selection criteria. Nonagreements were discussed until consensus was reached. Papers were included if they (1) studied (potential) patients (with or without an established diagnosis or condition), relatives (or other nonrelated informal caregivers), or health care professionals; (2) studied the effects of Web-based patient narratives (real experiences or fictional stories; collections or single narratives; presentation as text or audio or video clips) that were generated scientifically (eg, through qualitative research methods and not just stories put selectively on the Web with a view to their human interest for marketing purposes); and (3) were quantitative studies such as surveys and questionnaires, observational studies, nonrandomized controlled trials (non-RCTs), RCTs, comparative effectiveness research, cohort studies, or longitudinal studies. We excluded studies that used qualitative study designs such as interview studies, focus groups, or ethnographic studies and studies that were neither published in English or German. Studies that used narratives that were not generated by a scientific method were also excluded (eg, unmoderated blogs or fora). Furthermore, we excluded studies published before 2000 and studies that examined narratives not Web-based. We made no restrictions on the inclusion of studies regarding content, context, length, or depth of the narratives. We decided to focus on Web-based narratives as we felt the range would have been too broad to allow for meaningful comparisons had we included narratives available in different media (books, leaflets, newspapers, etc).

### Quality Assessment

A protocol was written about all the steps of data collection and analysis, including selection of studies and extraction of content. Researchers were trained in applying the predefined selection criteria. Overall, 3 researchers reviewed and assessed all studies (DD, AG, and MH), whereas nonagreements were discussed with a fourth independent expert (NB). Evaluation tools designed for conventional systematic reviews typically assess the quality of RCTs. However, the diversity of research designs and outcome measures of the included studies required the use of a tool that is able to systematically appraise disparate evidence stemming from different study types. Therefore, 2 authors (DD and AG) independently performed an assessment of the quality of the included studies using the checklist proposed by Hawker et al [22]. This validated checklist consists of nine evaluation sections: abstract and title, introduction and aims, method and data, sampling, data analysis, ethics and bias, results, transferability or generalizability, and implications and usefulness. Each section was assessed by giving a score ranging from 1 to 4 (4=good, 3=fair, 2=poor, and 1=very poor), resulting in a potential score range of 9 to 36. Similar to the Appraisal of Guidelines for Research and Evaluation II instrument [23], we calculated sum scores for each section and an overall score, scaled as a percentage of the maximum possible score over all sections:

obtained score−minimum possible score/maximum possible score−minimum possible score×100

### Data Extraction

A data elicitation form was developed and applied systematically to all publications included in the review by 1 author (DD). The form includes information about background characteristics (authors, year of publication, and location), study characteristics (aim, sample size, participants, and study design), narrative (type of narrative and degree of exposure), study measures (attitudes and beliefs, psychometric scales, and preferences), and a summary of findings.

### Data Synthesis

We extracted study results as they were reported in the results section of the publications. The analysis was based on the comparison of study details using descriptive statistics and text. The analysis was mainly focused on the identification of similarities and differences between the findings of the individual studies. As the study aims, designs, and findings were too heterogeneous, a meta-analysis was not conducted.

The specific outcomes of the studies were grouped using the taxonomy proposed by Shaffer and Zikmund-Fisher [3]. As several of the included studies provided few details about the content of the narratives, the studies were grouped around the purpose of the narrative. According to Shaffer and Zikmund-Fisher [3], five different purposes of narratives can be described. As most of the studies focus on (potential) patients rather than on relatives or health care professionals, the Shaffer and Zikmund-Fisher [3] taxonomy is suitable for our review. The purposes and their possible outcomes as proposed by Shaffer and Zikmund-Fisher [3] are described in Table 1.

**Table 1:** Purposes of narratives.

Purpose	Possible outcomes
Inform	Increased knowledge Improved affective forecasting
Engage	Greater engagement Greater transportation (increased depth of processing) Greater time spent with materials
Model behavior	Increased participation in health care decisions Increased shared decision making Altered behavioral intentions Increased uptake of target behaviors
Persuade	Altered behavioral intentions Increased uptake of target behaviors
Comfort	Reduced psychological distress Reduced anxiety

The definition of *effective* and *preference-sensitive* decisions proposed by Wennberg et al [24,25] was applied to assign the outcomes of the included studies to *risks* and *benefits* categories: outcomes were assigned to the *risk* category when they were *preference sensitiv*e. In *preference-sensitive* decisions, the best decision for an individual is unclear because of two reasons: the evidence for specific treatments is inadequate and firm conclusions about risk-to-benefit ratios cannot be drawn and the risk-to-benefit ratio might be clear, but it depends on the patients’ values [24,25]. Outcomes were assigned to the *benefit* category when they were *effective* following the definition by Wennberg et al [24,25]. In these cases, the best decision is clear to practitioners and patients. The clinical evidence of harms and benefits is known, and compared with the benefits, the harms are minimal. In *effective* decisions, there is a widespread consensus among clinicians and patients about known and favorable risk-to-benefit ratios [24,25]. The outcomes of the included studies were assigned to a *no-effect* category when the corresponding studies reported experimental conditions inferior to the control group or no statistically significant effects (significance level chosen by the individual study). In descriptive studies, thresholds such as significance levels are not available. Therefore, outcomes of descriptive studies that were mentioned by ≤50% of the participants were also assigned to the *no-effect* category.

## Results

### Literature Search

**Figure 1:**
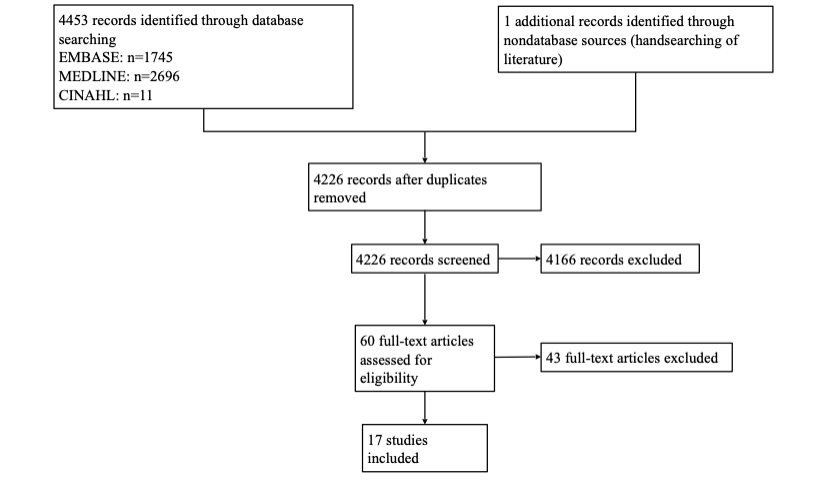
Study flowchart.

Our search strategy identified 4226 documents. Of these, 60 documents potentially fulfilled the inclusion criteria of the study and were assessed in full text. After assessing the full texts, 43 more studies were excluded for specific reasons, including, eg, the study did not focus on systematically generated narratives or the narratives were not Web-based. There were 95.50% (4036/4226) agreements among the raters. Finally, 17 studies were included in the analysis (Figure 1).

### Description of Included Studies

The studies were taken from Germany (n=5), the United States (n=6), the United Kingdom (n=4), the Netherlands (n=1), and Switzerland (n=1) and covered the period from 2000 to 2018 (Table 2). They investigated 10 different Web sources. The Web source and their specifications are shown in Multimedia Appendix 2.

**Table 2:** Characteristics of the included studies.

Authors (year)	Country applied	Sample size	Name of database	Degree of exposure to the narrative (eg, length of stay on a website)
Aardoom et al [26] (2014)	Netherlands	311	Proud2Bme	Mean time in months since first website visit: 19.8. Participants indicating to visit the website every day to several times a day: 189/247 (76.5%)
Allam et al [27] (2015)	Switzerland	157	ONESELF	Mean visits to the website: 53.68 (SD 93.07)
Betsch et al [14] (2011)	Germany	385 (study 1: 72; study 2: 313)	Online bulletin board	NR^a^ (paper-and-pencil version of an online bulletin board)
Betsch et al [15] (2013)	Germany	458	Simulated website similar to the website *patientslikeme*	Mean time in minutes to complete the study: 9.94 (SD 3.49)
Brunette et al [28] (2015)	United States	39	Let’s Talk About Smoking	NR
Engler et al [18] (2016)	Germany	23	DIPEx^b^	NR
Giesler et al [29] (2017)	Germany	212	DIPEx	Mean time in minutes on the intervention website: 42.21 (SD 45.64, median 26)
Newman et al [30] (2009)	United Kingdom	37	DIPEx	NR (paper-and-pencil survey)
Shaffer et al [31] (2013)	United States	302	Web decision aid	Mean time in minutes on the intervention website: 5.38 (SD 2.37); mean time in minutes on the control website: 4.92 (SD 2.03)
Shaffer et al [32] (2013b)	United States	56	Web decision aid	Mean time in seconds on different pages with text narratives: 5.00-67.28; mean time in seconds on different pages with video narratives: 15.11-117.19
Shaffer et al [33] (2014)	United States	200	Web decision aid	Length of narrative video: approximately 1 hour
Schweier et al [34] (2014)	Germany	571	lebensstil-aendern	Website usage in the intervention group: 46.1% (119/258); website usage in the control group: 7.0% (22/313)
Snow et al [35] (2016)	United Kingdom	88	DIPEx	Expected time to complete the module: 20 min. No time limits were set. Participants could watch the videos multiple times
Sullivan et al [36] (2018)	United States	2125 (study on acid reflux: 1070; study on high blood pressure: 1055)	Simulated prescription drug websites	All participants were exposed to the video. Participants that viewed the entire video: 94.86% (1015/1070) (acid reflux) and 98.66% (1041/1055) (high blood pressure). Participants that replayed the video: 7.5% (acid reflux) and 6.8% (high blood pressure)
Winterbottom et al [37] (2012)	United Kingdom	1694 (study 1: 578; study 2: 1116)	Web decision aid	NR
Wise et al [38] (2008)	United States	353	Comprehensive Health Enhancement Support System	No directives for the frequency of website use was given. Access to the website was given for four months.
Yaphe et al [39] (2000)	United Kingdom	309	DIPEx	NR

^a^Not reported.

^b^DIPEx: Database of Individual Patients’ Experiences.

Sample sizes of the studies ranged from 23 to 2125 (samples of the following substudies were combined: Betsch et al [14], Sullivan et al [36], and Winterbottom et al [37]) with a median of 302 per study. Most of the included studies focus on the effects on (potential) patients. Only one study that met our inclusion criteria focused on (future) health care professionals (medical students) [35]. The measures of the studies were (1) general perceptions of patient narratives, including patients’ expectations and learning experiences [18,30], self‐help and use of patients’ stories [39], and empowering processes and outcomes experienced by website participants [26]; (2) effects of narratives on patients’ and health care professionals’ behavior, including health care participation [38]; information search, treatment intentions, and decision satisfaction [32,33]; self-efficacy coping with cancer and patient competence [29]; physical activity [27,34]; health care utilization and medication overuse [27]; and performance in examinations [35]; and (3) decision making about dialysis modality [37], tobacco cessation treatment [28], vaccination [14,15], reflux and blood pressure drugs [36], and early-stage breast cancer [31,33].

The degree of exposure to the narrative was reported by 11 out of 17 studies. The reporting included measures such as self-reporting regarding frequency of website visits [26,34], mean visiting times of the websites [27] or mean times spent on the corresponding websites [15,29-31], the length of the narrative videos or expected study length [33,35], the number of participants exposed to the narratives [36], and the timespan for which participants had access to the corresponding websites [38].

The mean score of the quality assessment was 84.5 (range 61-100). The main issues were concerning appropriate sampling strategies [14,30,37], ethical issues regarding the relationship between researchers and participants [14,15,32,38,39], and the transferability of the study findings to a wider population [14,30,32,33,37,39] (Table 3). Among all the experimental studies, allocation concealment and study blinding were not adequately reported.

**Table 3:** Quality assessment of included studies.

Authors (year)	Abstract and title^a^	Introduction and aims^a^	Method and data^a^	Sampling^a^	Data analysis^a^	Ethics and bias^a^	Results^a^	Transferability or generalizability^a^	Implications and usefulness^a^	Scaled overall score^b^
Aardoom et al [26] (2014)	8	8	8	6	8	8	8	7	6	90.6
Allam et al [27] (2015)	8	8	8	8	8	8	8	8	8	100
Betsch et al [14] (2011)	8	8	8	4	7	2	7	5	6	68.4
Betsch et al [15] (2013)	8	8	8	8	7	2	7	8	8	85.2
Brunette et al [28] (2015)	8	8	8	8	8	8	8	8	8	100
Engler et al [18] (2016)	8	6	7	8	5	8	8	6	6	81.5
Giesler et al [29] (2017)	8	8	8	8	7	8	8	8	8	98
Newman et al [30] (2009)	8	8	4	4	4	8	6	4	6	63
Shaffer et al [31] (2013)	8	8	8	6	8	8	8	8	6	92.6
Shaffer et al [32] (2013b)	8	8	8	5	6	2	6	5	8	70.4
Shaffer et al [33] (2014)	8	7	7	6	5	6	6	4	8	72.1
Schweier et al [34] (2014)	8	8	8	8	8	8	8	8	8	100
Snow et al [35] (2016)	8	8	8	8	8	7	8	8	8	98
Sullivan et al [36] (2018)	8	8	8	8	8	8	8	8	8	100
Winterbottom et al [37] (2012)	7	8	8	4	4	8	8	5	6	74.1
Wise et al [38] (2008)	8	8	8	7	7	2	8	6	8	81.5
Yaphe et al [39] (2000)	8	7	6	7	4	2	6	4	7	61

^a^Sum score ranging from 2 to 8.

^b^Scaled overall score ranging from 0 to 100.

### Description of Study Methodologies

The study design varied among the included studies (Table 4): nine used experimental designs, including four RCTs [29-35], and seven used factorial designs [14,15,32,33,36,37], two were descriptive cross-sectional survey studies [26,39], two were mixed method studies [18,30], one was a pre-post pilot study [28], and one was a secondary analysis [38]. Only one study [34] used an intention-to-treat analysis. Furthermore, 6 studies were informed by a theoretical framework, including the social learning theory [34,38], empowerment construct [26], social support features and gamification elements [27], theory of planned behavior [28], and a self-developed taxonomy of patient stories that provides a framework [31].

**Table 4:** Description of study methodologies.

Authors (year)	Study design	Measures (attitudes, psychometric scales, preferences, behavior, etc)	Type of participants
Aardoom et al [26] (2014)	Cross-sectional (descriptive online survey)	Eating psychopathology, general empowerment, symptom duration, treatment status, and user activity	Website visitors who indicated having eating problems
Allam et al [27] (2015)	5-arm parallel randomized controlled trial	Physical activity, health care utilization, medication overuse, empowerment, and rheumatoid arthritis knowledge	Individuals diagnosed with rheumatoid arthritis
Betsch et al [14] (2011)	Factorial between-subjects design	Perceived risk of side effects and vaccination intentions	Students
Betsch et al [15] (2013)	Factorial between-subjects design	Perceived risk, vaccination intention, and subjective numeracy	General population
Brunette et al [28] (2015)	Pre-post pilot study	Use of cessation treatment	Individuals smoking ≥4 cigarettes
Engler et al [18] (2016)	Mixed method approach including log file analyses, descriptive survey data analyses, and thematic analysis of focus group discussions (only quantitative results are extracted).	Attitudes toward health-related websites in general, perception of krankheitserfahrungen.de in particular	Individuals diagnosed with colorectal, breast, or prostate cancer
Giesler et al [29] (2017)	Randomized two-group between-subjects design with repeated measures.	Coping self-efficacy and patient competencies	Individuals diagnosed with colorectal cancer
Newman et al [30] (2009)	Mixed method. The study involved three stages: (1) focus groups guided the development of a descriptive questionnaire, (2) the questionnaire was modified, and (3) a sample of outpatients was asked to complete the questionnaire. (Only quantitative results are extracted.)	Attitudes toward the website	Individuals diagnosed with an inflammatory rheumatologic condition
Shaffer et al [31] (2013)	Factorial design	Information search, treatment intentions, and decision satisfaction	Women from the general population who were not pregnant and without a breast cancer history
Shaffer et al [32] (2013b)	Factorial design	Treatment preference	Women from the general population who were not pregnant and without a breast cancer history
Shaffer et al [33] (2014)	Factorial design	Treatment preference	Women from the general population who were not pregnant and without a breast cancer history
Schweier et al [34] (2014)	Sequential controlled trial	Diagnosis, BMI, baseline behavior for physical activity, eating routine, exercise frequency and attention paid to healthy diet, and improvements in physical activity and eating behavior	Individuals diagnosed with coronary heart disease
Snow et al [35] (2016)	Exploratory randomized controlled trial	Knowledge demonstration and clinical examination with a simulated patient	Medical students
Sullivan et al [36] (2018)	Factorial design	Risk perception	Individuals with self-reported acid reflux or high blood pressure
Winterbottom et al [37] (2012)	Factorial design	Hypothetical treatment choice	Students
Wise et al [38] (2008)	Secondary analysis	Health care participation and online information use	Women diagnosed with breast cancer
Yaphe et al [39] (2000)	Cross-sectional (descriptive survey study)	Whether and how patients’ stories are collected and used	Self-help groups

### Outcomes of Studies

Table 5 describes the effect directions of the outcomes of the included studies. The outcomes are grouped along the taxonomy from Shaffer and Zikmund-Fisher [3]. Most studies reported more than one outcome. Therefore, the number of outcomes is higher than the number of included studies.

**Table 5:** Effects of narratives on outcomes taxonomy.

Taxonomy, outcome, authors (year)			Effect direction		
			Risk	No effect	Benefit
**Inform**					
	**Competence**				
		Giesler et al [29] (2017)	N/A^a^	X^b^	N/A
		Snow et al [35] (2016)	N/A	N/A	X
	**Knowledge**				
		Allam et al [27] (2015)	N/A	X	N/A
		Engler et al [18] (2016)	N/A	N/A	X
**Engage**					
	**Empowerment**				
		Aardoom et al [26] (2014)	N/A	N/A	X
		Allam et al [27] (2015)	N/A	N/A	X
	**Length of information search**				
		Shaffer et al [31] (2013)	N/A	N/A	X
		Shaffer et al [32] (2013b)	N/A	N/A	X
	**Sharing experiences**				
		Engler et al [18] (2016)	N/A	N/A	X
		Newman et al [30] (2009)	N/A	N/A	X
		Yaphe et al [39] (2000)	N/A	N/A	X
**Model behavior**					
	**Eating behavior**				
		Schweier et al [34] (2014)	N/A	X	N/A
	**Health care utilization**				
		Allam et al [27] (2015)	N/A	N/A	X
		Wise et al [38] (2008)	N/A	N/A	X
	**Medication overuse**				
		Allam et al [27] (2015)	N/A	N/A	X
	**Physical activity**				
		Allam et al [27] (2015)	N/A	N/A	X
		Schweier et al [34] (2014)	N/A	X	N/A
**Persuade**					
	**Risk judgments**				
		Betsch et al [14] (2011)	X	N/A	N/A
		Betsch et al [15] (2013)	X	N/A	N/A
		Sullivan et al [40] (2018)	X	N/A	N/A
	**Treatment decisions**				
		Betsch et al [14] (2011)	X	N/A	N/A
		Betsch et al [15] (2013)	X	N/A	N/A
		Brunette et al [28] (2015)	N/A	N/A	X
		Shaffer et al [33] (2014)	N/A	X	N/A
		Winterbottom et al [37] (2012)	X	N/A	N/A
**Comfort**					
	**Confidence**				
		Shaffer et al [31] (2013)	N/A	N/A	X
		Snow et al [35] (2016)	N/A	N/A	X
	**Self-efficacy**				
		Giesler et al [29] (2017)	N/A	X	N/A

^a^Not applicable.

^b^Each X represents an individual study reporting statistically significant risks, no significant effects or statistically significant benefits.

#### Provide Information

Giesler et al [29] investigated patient competence, including self-regulation, effective coping with emotional distress, explicit dealing with cancer threat, and low avoidance, as a secondary outcome in their study. They reported no significant differences between the intervention and control groups. Snow et al [35] examined the effect of patient narratives describing their colposcopy on fifth-year medical students’ proficiency in standard examinations. They reported a significantly better performance in the examination compared with the control group that viewed a clinician describing the procedure (odds ratio [OR] 2.7, 95% CI 1.2-6.1; *P*=.02).

Allam et al [27] reported no significant improvements in the knowledge of rheumatoid arthritis. It should be noted that the initial level of the control group was significant. A study among cancer patients testing narrative cancer modules on the website *krankheitserfahrungen* found that 72% (40/56) agreed or strongly agreed that the internet is supportive to understanding what physicians tell them [18].

#### Engage

A study by Aardoom et al [26] reported that the exchange of information, finding recognition, sharing experiences with others, and feeling better informed were the most often reported empowering processes and outcomes. The authors concluded that online sources where individuals can share their experiences are promising strategies for successful electronic health (eHealth) initiatives such as *Proud2Bme*. A 5-arm parallel RCT found that levels of empowerment changed over time in study groups having access to online social support (beta=2.59; *P*=.03) or gamified experiences of a website (beta=2.29; *P*=.05) [27].

Participants viewing narratives relating how a patient makes her decision were found to spend more time searching for information regarding breast cancer (narrative condition, mean 5.38 min, SD 2.37, vs no narrative condition, mean 4.92 min SD 2.03 [31]; narrative condition, mean 39.88 min, SD 15.62, vs no narrative condition, mean 35.08 min, SD 16.09 [32]). Furthermore, Shaffer et al [31] reported that participants who viewed narratives containing experiences regarding diagnosis, treatment, or complications with early breast cancer treatments showed greater abilities to imagine how it might be to experience these treatments (imagine a mastectomy in the no narrative condition, mean 4.46, SD 1.21, vs imagine a mastectomy in the narrative condition, mean 4.69, SD 1.02, *t*=1.72; *P*=.04; imagine a lumpectomy with radiation in the no narrative condition, mean 4.44, SD 1.19, vs imagine a lumpectomy with radiation in the narrative condition, mean 4.72, SD 0.94, *t*=2.19; *P*=.01; measured on a 9-point Likert scale).

Findings showed that learning about other peoples' health-related experiences is relevant and helpful [18,30]. Furthermore, patients’ stories collected by DIPEx are frequently included in interviews or articles for group newsletters, newspaper articles, or media broadcasts by voluntary organizations [39]. Engler et al [18], eg, reported that 76% (43/56) of their participants agreed that it can be helpful to witness the health-related experiences of others. However, some of the younger participants in the study by Newman et al [30] reported that the site did not cover experiences of younger patients. The participants highlighted the importance of incorporating current and accurate information. Some participants were concerned that the site might be depressing to patients with a new diagnosis [30].

#### Model Target Behaviors

A statistically significant positive effect on physical activity was reported by Allam et al [27]. In contrast, Schweier et al [34] did not find significant effects on physical activity and eating behavior changes. Health care utilization and medication overuse decreased according to the findings of one study [27]. Furthermore, one study investigated the effects of Web-based narratives and didactic information on health care participation [38]. This study reported positive effects of an eHealth program with narratives (audiovisual and text; beta=.123; *P*<.01) and didactic information (text only; beta=.104; *P*<.05) on health care participation. Health care participation was measured on a 7-item, 5-point response scale. These effects were reported to be significantly greater for African Americans.

#### Persuade

A total of six studies investigated the effects of narratives on risk judgments [14,36] and treatment decisions, including hypothetical treatment choices between a lumpectomy with radiation or a mastectomy [33], vaccination intentions [14,15], hypothetical dialysis modalities [37], and cessation treatment [28].

Furthermore, two studies [14,15] focused on the effects of statistical and/or narrative information on vaccination decisions. Betsch et al [14] showed that the perceived risk of vaccination increases the more the narratives report adverse events (*F*_2,58_=3.852; *P*<.05; η^2^=0.12), and if adverse events are reported in a highly emotional manner (mean 15.33, SD 9.27 vs mean 17.52, SD 11.00; *F*_1,297_=4.197; *P*<.05; η^2^=0.01). Furthermore, they showed that the intention for vaccination decreases when the number of narratives increases (*F*_2,58_=5.729; *P*<.01; η^2^=0.17), which is partially mediated by an increased perception of risk [14]. Two years later, the same research group published results from a similar setting, which point in the same direction [15]. Sullivan et al [36] investigated the influence of videos on consumers’ knowledge, perceptions, and behavioral intentions. Participants were randomly assigned to 1 of 10 fictitious prescription drug websites. The video type (patient testimonial, informational video describing the mechanism of action, or none) and whether the video included drug risks was manipulated on each website. They found that participants who were exposed to any of the videos were less likely to recognize drug risks that were presented only on the website text. Videos that included risk information overall led to increased risk recognition. However, in some risk recognition measures such as risk of fracture, risk of special liver tests, or risk of angioedema, risk recognition decreased for risks that were not presented in the videos but risk information was always present in the website text. Furthermore, the study found no significant effects of risk prominence and type of video condition on physician interaction and search intentions on the internet.

In addition, one pilot study investigated the effect of a website that aimed to engage smokers in a cessation treatment [28]. Among 38 participants who used the website, 18 participants (47%) became abstinent for at least one day, 7 (18.4%) became abstinent for 7 or more days, and 4 (11%) became continuously abstinent. Winterbottom et al [37] demonstrated that hypothetical dialysis treatment choices presented as a patient narrative were more likely to be chosen by the participants than presented by a doctor (both using actors). Another study [33] found no differences in preferences for surgical treatments between women who watched videos that included narratives compared with those who watched a control video.

#### Provide Comfort

Snow et al [35] reported that students in the narrative condition reported significantly more confidence in comfort with patients’ emotions (OR 6.4, 95% CI 2.7-14.9; *P*<.001). The study by Shaffer et al [31] compared participants who viewed experience narratives with those not viewing experience narratives. They demonstrated increased confidence in the experience narratives condition regarding the ability to make an informed choice (mean 3.77, SD 0.90 vs mean 4.01, SD 0.84; *t*=2.33; *P*<.01), to be more thorough in considering relevant factors (mean 4.07, SD 0.73 vs mean 4.21, SD 0.64; *t*=1.72; *P*<.04), to be more confident in the awareness of relevant factors (mean 3.29, SD 0.95 vs mean 3.53, SD 0.90; *t*=2.21; *P*<.01), and to be more satisfied with their decision-making process (mean 3.76, SD 0.81 vs mean 3.95, SD 0.77; *t*=2.08; *P*<.02).

Giesler et al [29] evaluated the colorectal cancer module of the German DIPEx website with regard to coping self-efficacy as the primary outcome and patient competencies as the secondary outcome. The study results did not support the authors' hypothesis that the website increases self-efficacy for coping with cancer or patient competencies such as self-regulation or managing emotional distress at 2 and 6 weeks after baseline.

## Discussion

### Principal Findings

There is an increasing number of Web-based sources containing research-based, systematically generated accounts of patient illness and health experiences. Although the evidence on the persuasiveness of narrative information on individuals’ decision making was reviewed over a decade ago [4], we present, to our knowledge, the first systematic review about the effects of Web-based patient narratives on patients, relatives, or health care professionals.

Our review revealed several beneficial effects for patients and health care professionals. Web-based narratives are an effective way of teaching to improve knowledge and confidence for students as well as for patients [18,35]. Furthermore, research indicates that patients perceive other patients’ health experiences as relevant and helpful [18,30]. This finding points to the importance of the quality of health-related information [41]. Compared with the health-related information and experiences on general social media sites, academic research–based patient narratives might be less susceptible to challenges for the quality of health-related information through, eg, spamming, intentional misspelling, or actuality of information [41]. Several quality measures to evaluate the quality of Web-based health information are available [42,43].

Another identified benefit is that participants viewing narratives that contain information on how patients make decisions result in longer search times for information [31,32]. This effect can be a resource to increase, eg, patients’ health literacy. However, Shaffer et al [32] also reported that transcripts of the patient videos caused the opposite effect. Participants confronted with text-based narratives spent approximately five fewer minutes for information search. Researchers and health care professionals using patient narrative databases should be aware that the format of patient stories might be similarly important as the content in determining their effect on medical decision making [32].

On the basis of the findings of this review, it remains unclear whether patient narratives can influence patients’ target behavior. The results regarding physical activity are equivocal [27,34]. Narratives led to an increase of health care participation and decreased unnecessary health care utilization as well as medication overuse [27,38].

Even though we identified several benefits of patient narratives on the different purposes of narratives, overall, there is little evidence for the effects of Web-based patient narratives in a positive or negative way. The total number of studies we included in the review is small, which is especially challenging in the light of the heterogeneity regarding the sampled population, the study aims, and the heterogeneity of the narratives in itself. Furthermore, the purposes of the narratives presented on the different websites vary considerably from each other. However, patient narratives are not homogenous and have to be evaluated in their context with regard to content, purpose, and patients’ evaluative expression, such as expressions of (dis)satisfaction with processes of health care decision making [3]. We concur with the position of Shaffer and Zikmund-Fisher [3] that the role of narratives can only be fully understood if operational definitions of narratives are sufficient. Furthermore, there is a need for more theoretical conceptions about the impact of narratives on specific outcomes. We found that only 6 out of 17 studies were guided by a theoretical framework [31,32,34,37-39]. The lack of theoretical frameworks might partially be explained by the fact that research on narratives on certain patient-relevant outcomes is a relatively new field with a range of potentially relevant outcomes. Giesler et al [29], eg, found no significant differences at 2 weeks between an intervention group that had immediate access to the colorectal cancer module of the DIPEx website and a control group with regard to self-efficacy for coping with cancer and patient competence. Study participants in the intervention group visited the website on average for 42.21 min with 3.31 mean number of sessions. Such findings do not necessarily indicate that there is no effect of patient narratives. It rather highlights that the specific outcomes that were investigated in this study and in the study-specific setting were not significant. Other psychological measures on self-efficacy for coping or on patient competence may have led to different results. Another indication for the overall little evidence is that the effects of several studies reporting significant outcomes are rather small or are only significant under specific experimental conditions. For example, the difference between the mean search time for information regarding breast cancer reported by Shaffer et al [32] is 0.46 min, with an average search time of 5.38 min in the intervention group and 4.92 min in the control group.

Almost one-third of the included studies used study samples that cast doubts whether the results can be generalized to broader patient populations [14,30,32,37], Clear definitions of the basic population and appropriate sampling strategies would be desirable in future studies. Schlesinger et al [44] demonstrated that a rigorous collection of patient narratives can also be incorporated into large patient experience surveys.

At the same time, narratives can bear potential risks in preference-sensitive decisions [24]. There is a growing body of evidence on the effect of narrative bias [4,14,15], where narrative information can override risk judgments. This effect can even occur when base rate information is presented in addition [15]. Narratives are widely used in patient decision aids [45]. Furthermore, it is likely that narratives are used by other patients as decision support tools, although they are not explicitly declared as decision aids. Decision aids are evidence-based tools with an aim to support patients in a value-sensitive way to make specific health care choices [46]. Narratives may reduce the effectiveness of decision aids by presenting unbalanced information or by overriding decision-relevant information through characteristics of the narrator [4,45]. For example, a study conducted by Khangura et al [45] indicates that patient narratives in decision aids were more likely to portray patients that were satisfied with the outcome of their treatment decision. This points to the importance of including disclaimers that highlight the potential for biases in patient narratives [15]. Furthermore, this highlights the need for a careful selection of the presented stories on patient narrative databases by the corresponding research teams in charge for the databases to present a balanced picture of the whole spectrum of health experiences [47]. This might be especially important in narratives about health conditions where public opinions are mixed and biases might be suspected.

Qualitative studies focusing on how individuals use and value personal health-related experiences [10], decision making regarding prostate cancer [11], or information needs of patients with cancer and their views of internet-based health information [12] indicate improvements in decision making [10,11] and in meeting information needs [12]. These findings are not completely in line with our review of quantitative studies. How can this difference be explained? Both approaches study different phenomena. The foundations of the qualitative paradigm are interpretivism and constructivism, where multiple socially constructed realities are investigated [48,49]. On the contrary, the quantitative approach is based on positivism, which assumes that phenomena can be represented by empirical indicators that represent the one and only truth [49]. It can be speculated that the qualitative findings rather represent the *lived experiences* of patients’ decision making, whereas the quantitative results represent quantitative measures of the decision-making processes.

### Limitations

Our study has several limitations. First, we searched only for papers published in journals, and only in English or German. Papers that were published in books or reports are often not indexed in the databases we have chosen for our search strategy and are therefore not included. Therefore, we may have missed some studies published in languages or places outside our scope. Second, we reviewed only published studies regarding patient narratives. Therefore, we may potentially be confronted with a publication bias in such a way that, eg, negative study results were not published. Third, we decided to include only studies that focused on Web-based narratives and that were generated through a research methodology. Although we have done so to ensure comparability among the studies, we also acknowledge that this decision has led to an exclusion of several studies that investigated the effects of non–Web-based narratives or generated in an unstructured, non–research-based way, eg, in chatrooms or fora. Narratives are valuable resources for the narrators themselves, for other patients and their relatives, and for health care professionals and researchers. Despite the limitations, our findings might be helpful for health care professionals and researchers to understand the possible effects of narratives in health care settings.

### Conclusions

In total, we found 17 studies on the effects of Web-based patient narratives. The effects of narratives were classified by purpose—inform, engage, model behavior, persuade, and comfort—using the taxonomy provided by Shaffer and Zikmund-Fisher [3]. Overall, patient narratives seem to be a promising means to improve knowledge of health care professionals and patients. Learning about other patients’ experiences is perceived as supportive and relevant. Furthermore, they can positively influence patient empowerment. There is some evidence of beneficial effects on some outcomes, such as information search and the modeling of target behavior such as physical activity, health care participation, and medication overuse. The narratives used in the studies are characterized by considerable heterogeneity, and the investigated outcomes are hardly comparable among each other, which makes an overall judgment difficult.

## Appendix

Multimedia Appendix 1 Search terms.

Multimedia Appendix 2 Investigated databases and descriptions.

## Abbreviations

DIPEx: Database of Individual Patients’ Experiences
eHealth: electronic health
OR: odds ratio
RCT: randomized controlled trial
