# Supplementary material for: Risks and Benefits of Web-Based Patient Narratives: Systematic Review
Source: J Med Internet Res. 2020 Mar 26;22(3):e15772. doi: 10.2196/15772 (PMC7146251; doi:10.2196/15772)
Supplement: Multimedia Appendix 1 [file jmir_v22i3e15772_app1.docx]

**Appendix I: Search Terms**

**Pubmed (via OVID)**

exp narration/ or exp personal narratives as topic/ or exp medicine, narrative/ or exp anecdotes as topic/ or (narrati$ or anecdote$ or testimonial$).ti,ab. or (health and talk$).ti,ab. or (story adj3 tell$).ti,ab. or ((patient) adj3 (narrati$ or experience$)).ti,ab.

AND

exp internet/ or exp medical informatics/ or (internet$based or online or web$based or database or social$media or DIPEx or digital health or website$).ti,ab.

AND

exp health/ or exp health information exchange/ or exp delivery of health care/

AND

exp patients/ or exp health personnel/ or exp caregivers/ or exp proxy/ or exp family/ or patient$.ti,ab. or (health adj3 personnel).ti,ab. or (caregiv$ or family or informal caregiv$).ti,ab. or (family adj3 member).ti,ab.

AND

(exp RANDOMIZED CONTROLLED TRIAL/ OR exp CONTROLLED CLINICAL TRIAL/ OR exp RANDOM ALLOCATION/ OR exp DOUBLE BLIND METHOD/ OR exp SINGLE BLIND METHOD/ OR exp clinical trial/ OR exp PLACEBOS/ OR exp RESEARCH DESIGN/ OR exp COMPARATIVE STUDY/ OR exp EVALUATION STUDIES/ OR exp FOLLOW UP STUDIES/ OR exp PROSPECTIVE STUDIES/ OR (clin$ adj25 trial$).ti,ab. OR ((singl$ or doubl$ or trebl$ or tripl$) adj25 (blind$ or mask$)).ti,ab. OR (placebo$ OR random$).ti,ab. OR (control$ or prospectiv$ or volunteer$).ti,ab.) or exp health care surveys/ or exp Questionnaires/ or exp cohort studies/ or exp longitudinal studies/ or (survey$ or questionnaire$ or cohort stud$ or longitudinal stud$).ti,ab.

**Pubmed (via Pubmed)**

(((((((((((((((narration[MeSH Terms]) OR personal narratives as topic[MeSH Terms]) OR medicine, narrative[MeSH Terms]) OR anecdotes as topic[MeSH Terms]) OR narrati*[Title/Abstract]) OR anecdote*[Title/Abstract]) OR testimonial*[Title/Abstract]) OR health talk*[Title/Abstract]) OR story tell*[Title/Abstract]) OR patient* narrati*[Title/Abstract]) OR patient* experience*[Title/Abstract]))

AND

((((((((((((internet[MeSH Terms]) OR medical informatics[MeSH Terms]) OR internet based[Title/Abstract]) OR internet-based[Title/Abstract]) OR web based[Title/Abstract]) OR web-based[Title/Abstract]) OR database[Title/Abstract]) OR social media[Title/Abstract]) OR social-media[Title/Abstract]) OR DIPEx) OR digital health[Title/Abstract]) OR website*[Title/Abstract]))

AND

(((health[MeSH Terms]) OR health information exchange[MeSH Terms]) OR delivery of health care[MeSH Terms]))

AND

(((((((((((patients[MeSH Terms]) OR health personnel[MeSH Terms]) OR caregivers[MeSH Terms]) OR proxy[MeSH Terms]) OR family[MeSH Terms]) OR patient*[Title/Abstract]) OR health personnel[Title/Abstract]) OR caregiv*[Title/Abstract]) OR informal caregiv*[Title/Abstract]) OR family[Title/Abstract]) OR family member*[Title/Abstract]))

AND

((((((((((((((((((((((((((((randomized controlled trial[MeSH Terms]) OR controlled clinical trial[MeSH Terms]) OR random allocation[MeSH Terms]) OR double blind method[MeSH Terms]) OR single blind method[MeSH Terms]) OR clinical trial[MeSH Terms]) OR placebos[MeSH Terms]) OR research design[MeSH Terms]) OR comparative study[MeSH Terms]) OR evaluation studies[MeSH Terms]) OR follow up studies[MeSH Terms]) OR prospective studies[MeSH Terms]) OR clin* trial*[Title/Abstract]) OR ((singl*[Title/Abstract] OR doubl*[Title/Abstract] OR trebl*[Title/Abstract] OR tripl*)[Title/Abstract])) AND ((blind*[Title/Abstract] OR mask*)[Title/Abstract])) OR placebo*[Title/Abstract]) OR random*[Title/Abstract]) OR control*[Title/Abstract]) OR prospectiv*[Title/Abstract]) OR volunteer*[Title/Abstract]) OR health care surveys[MeSH Terms]) OR questionnaires[MeSH Terms]) OR cohort studies[MeSH Terms]) OR longitudinal studies[MeSH Terms]) OR survey*[Title/Abstract]) OR questionnaire*[Title/Abstract]) OR cohort stud*[Title/Abstract]) OR longitudinal stud*[Title/Abstract])

**EMBASE**

'narrative'/exp OR 'narrative medicine'/exp OR 'personal narrati*':ab,ti OR anecdot*:ab,ti OR testimonial*:ab,ti OR (health NEAR/3 talk*):ab,ti OR (story NEAR/3 tell*):ab,ti OR (patient NEAR/3 (narrati* OR experience*)):ab,ti

AND

'internet'/exp OR 'medical informatics'/exp OR 'internet?based':ab,ti OR 'online':ab,ti OR 'data?base':ab,ti OR 'web?based':ab,ti OR 'social?media':ab,ti OR 'dipex':ab,ti OR 'digital health':ab,ti OR 'website*':ab,ti

AND

'health'/exp OR 'health care'/exp OR 'health care delivery'/exp OR health:ab,ti OR health?care:ab,ti

AND

'patient'/exp OR 'health care personnel'/exp OR 'caregiver'/exp OR 'proxy'/exp OR 'family'/exp OR patient*:ab,ti OR (health NEAR/3 personnel):ab,ti OR caregiv*:ab,ti OR family:ab,ti OR 'informal caregiv*':ab,ti OR (family NEAR/3 member):ab,ti

AND

'randomized controlled trial'/exp OR 'controlled clinical trial'/exp OR 'randomization'/exp OR 'double blind procedure'/exp OR 'single blind procedure'/exp OR 'clinical trial'/exp OR 'placebo'/exp OR 'comparative study'/exp OR 'evaluation study'/exp OR 'follow up'/exp OR 'prospective study'/exp OR (('(clin*' NEAR/25 'trial*)'):ab,ti) OR '((singl*':ab,ti OR 'doubl*':ab,ti OR 'trebl*':ab,ti OR (('tripl*)' NEAR/25 '(blind*'):ab,ti) OR 'mask*))':ab,ti OR '(placebo*':ab,ti OR 'random*)':ab,ti OR '(control*':ab,ti OR 'prospectiv*':ab,ti OR 'volunteer*)':ab,ti OR 'health care survey'/exp OR 'questionnaire'/exp OR 'cohort analysis'/exp OR 'longitudinal study'/exp OR '(survey*':ab,ti OR 'questionnaire*':ab,ti OR 'cohort stud*':ab,ti OR 'longitudinal stud*)':ab,ti

**CINAHL (Ebscohost)**

(MH narratives OR TI ( (narrati* OR anecdote OR testimonial*) ) OR AB ( (narrati* OR anecdote OR testimonial*) ) OR TI ( (health AND talk*) ) OR AB ( (health AND talk*) ) OR TI (story N3 tell*) OR AB (story N3 tell*) OR TI ( ((patient) N3 (narrati* OR experience*)) ) OR TI (personal N3 narrati*) OR AB (personal N3 narrati*) OR AB (personal N3 narrati*))

AND

(MH internet OR MH medical informatics OR TI ( (internet*based OR online OR web*based OR database OR social*media OR DIPEx OR digital health or website*) ) OR AB ( (internet*based OR online OR web*based OR database OR social*media OR DIPEx OR digital health or website*) ))

AND

(MH health OR MH Health Care Delivery)

AND

(MH patients OR MH health personnel OR MH caregivers OR MH proxy OR MH family OR TI patient* OR AB patient* OR TI (health N3 personnel) OR AB (health N3 personnel) OR TI ( (caregiv* OR family OR informal caregiv*) ) OR AB ( (caregiv* OR family OR informal caregiv*) ) OR TI (family N3 member) OR AB (family N3 member))

AND

(MH Randomized Controlled Trials OR MH Clinical Trials OR MH Random Assignment OR MH Double-Blind Studies OR MH Single-Blind Studies OR MH Placebos OR MH Study Design OR MH Comparative Studies OR MH Evaluation Research OR MH Prospective Studies OR MH Surveys OR MH Questionnaires OR MH Nonexperimental Studies OR TI (clin* N25 trial*) OR AB (clin* N25 trial*) OR TI ((singl* OR doubl* OR trebl* OR tripl*) N25 (blind* OR mask*)) OR AB ((singl* OR doubl* OR trebl* OR tripl*) N25 (blind* OR mask*)) OR TI (placebo* OR random*) OR AB (placebo* OR random*) OR TI (control* OR prospectiv* OR volunteer*) OR AB (control* OR prospectiv* OR volunteer*) OR TI (survey* OR questionnaire* OR cohort stud* OR longitudinal stud*) OR AB (survey* OR questionnaire* OR cohort stud* OR longitudinal stud*))
