# Supplementary material for: Risks and Benefits of Web-Based Patient Narratives: Systematic Review
Source: J Med Internet Res. 2020 Mar 26;22(3):e15772. doi: 10.2196/15772 (PMC7146251; doi:10.2196/15772)
Supplement: Multimedia Appendix 2 [file jmir_v22i3e15772_app2.docx]

**Appendix II: Investigated databases and descriptions**

| **First author** | **Online source** | **Description of the online source** |
| --- | --- | --- |
|  |  |  |
| Engler et al. 2016 | DIPEx | Online information on people’s experiences with health and illness that have been systematically collected through qualitative interviews and analysed with rigorous qualitative research methods. |
| Giesler et al. 2017 |  |  |
| Newman et al. 2009 |  |  |
| Yaphe et al. 2000  Snow et al. 2016 |  |  |
|  |  |  |
| Shaffer et al. 2013a | Web decision aid | Video decision aid produced by Health Dialog that covers surgical options for early-stage breast cancer. |
| Shaffer et al. 2013b |  |  |
| Shaffer et al. 2014 |  |  |
|  |  |  |
| Aardoom et al. 2014 | Proud2Bme | Interactive e-community aimed at empowering individuals with symptoms of an eating disorder and promoting a positive body image and healthy lifestyle. |
|  |  |  |
| Allam et al. 2015 | ONESELF | Web-based intervention designed and operated for chronically ill patients with rheumatoid arthritis. |
|  |  |  |
| Betsch et al. 2011 | Simulated online bulletin boards | Simulated bulletin board with authentic postings from real online bulletin boards as stimulus material. |
| Betsch et al. 2013 |  |  |
|  |  |  |
| Brunette et al. 2015 | Let’s Talk About Smoking | Website that was designed to motivate smokers to quit by using evidence-based cessation treatment. |
|  |  |  |
| Schweier et al. 2014 | Lebensstilaendern | Non-restricted, no-cost, German language website that provides more than 1000 video, audio, and text clips from interviews with people with coronary heart disease and chronic back pain. |
|  |  |  |
| Wise et al. 2008 | CHESS | Comprehensive Health Enhancement Support System (CHESS) is developed by an interdisciplinary team of, healthcare clinicians and system engineers, health educators and communication specialists, computer programmer and. It provides didactic and narrative information about medical, practical and psychosocial issues. |
|  |  |  |
| Winterbottom et al. 2012 | Simulated online information | Online information about haemodialysis and continuous cycling peritoneal dialysis. |
| Sullivan et al. 2018 | Simulated prescription drug websites | Drug website advertising a fictitious drug. |
